# Social Isolation and Loneliness in Older Adults: A National Academies of Sciences, Engineering, and Medicine Report

**DOI:** 10.1093/geroni/igaa057.2511

**Published:** 2020-12-16

**Authors:** Colleen Galambos, James Lubben

## Abstract

Social isolation and loneliness (SIL) are serious yet underappreciated public health risks for many older adults (AARP, 2018a). Strong evidence suggests that, for older adults, social isolation and loneliness are associated with an increased likelihood of early death, dementia, heart disease, and more (AARP, 2018b, Holt-Lunstad and Smith, 2016). While all ages may experience SIL, older adults are at increased risk because they are more likely to face predisposing factors such as living alone, the loss of family or friends, chronic illness, and sensory impairments. Health care providers may be in the best position to identify older individuals who are at highest risk for SIL – individuals for whom the health care system may be the only point of contact with their broader community. The National Academies of Sciences, Engineering, and Medicine (NASEM) developed a consensus study report on this issue. This symposium presents the study recommendations. Dr. Holt-Lunstad examines the recommendations to develop a more robust evidence base for effective assessment, prevention, and intervention strategies for social isolation and loneliness. Dr. Galambos examines the recommendations to translate current research into health care practices and to improve awareness of the health and medical impacts of SIL. Dr. Lustig examines the recommendations to strengthen ongoing education and training and to strengthen ties between the health care system and community-based resources. Dr. Demiris examines the role of technology across all of these recommendations. Loneliness and Social Isolation Interest Group Sponsored Symposium

